# Reciprocal cooperation between unrelated rats depends on cost to donor and benefit to recipient

**DOI:** 10.1186/1471-2148-12-41

**Published:** 2012-03-29

**Authors:** Karin Schneeberger, Melanie Dietz, Michael Taborsky

**Affiliations:** 1 Behavioural Ecology Division, Institute of Ecology and Evolution, Bern, Switzerland; 2 Evolutionary Ecology Group, Leibniz Institute for Zoo and Wildlife Research, Berlin, Germany

## Abstract

**Background:**

Although evolutionary models of cooperation build on the intuition that costs of the donor and benefits to the receiver are the most general fundamental parameters, it is largely unknown how they affect the decision of animals to cooperate with an unrelated social partner. Here we test experimentally whether costs to the donor and need of the receiver decide about the amount of help provided by unrelated rats in an iterated prisoner's dilemma game.

**Results:**

Fourteen unrelated Norway rats were alternately presented to a cooperative or defective partner for whom they could provide food via a mechanical apparatus. Direct costs for this task and the need of the receiver were manipulated in two separate experiments. Rats provided more food to cooperative partners than to defectors (direct reciprocity). The propensity to discriminate between helpful and non-helpful social partners was contingent on costs: An experimentally increased resistance in one Newton steps to pull food for the social partner reduced the help provided to defectors more strongly than the help returned to cooperators. Furthermore, test rats provided more help to hungry receivers that were light or in poor condition, which might suggest empathy, whereas this relationship was inverse when experimental partners were satiated.

**Conclusions:**

In a prisoner's dilemma situation rats seem to take effect of own costs and potential benefits to a receiver when deciding about helping a social partner, which confirms the predictions of reciprocal cooperation. Thus, factors that had been believed to be largely confined to human social behaviour apparently influence the behaviour of other social animals as well, despite widespread scepticism. Therefore our results shed new light on the biological basis of reciprocity.

## Background

Cooperative behaviour is widespread among animals [1,2]. Theoretical models include both costs to the donor and benefits for the receiver as important parameters for cooperative behaviour to evolve [3-5]. While costs to the donor may be outweighed by indirect fitness benefits due to genetic similarity or relatedness with the receiver [2,3,6-9], cooperation between non-kin can evolve by mutualism or reciprocity [4,10,11]. Similar to Hamilton's rule explaining the evolution of cooperation by kin selection [3], costs and benefits play a crucial role also in the evolution of reciprocal cooperation [5]. However, their importance for an animal's decision to cooperate is little understood [12,13].

Reciprocal cooperation [10] among unrelated social partners has been described in a wide range of species, including mammals, birds and fish [11,14-20]. Over the past 40 years the conditions have been hotly debated under which reciprocal cooperation might emerge [4,10,11], however experimental tests of the effect of costs to the donor and benefits to the recipient on decisions to cooperate are scarce. To bridge this gap we manipulated both, the costs of the donor and potential benefit to the recipient in an iterated prisoner's dilemma game using unrelated female wild-type Norway rats (*Rattus norvegicus*).

**Figure 1:**
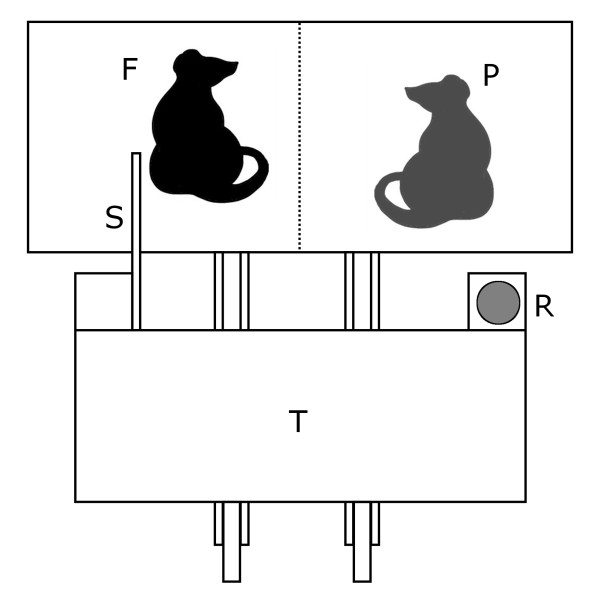
**Experimental setup**. The focal rat (black, F) can pull the tray (T) towards the cage with help of a stick (S) so that her social partner (grey, P) can reach the food reward (R).

Norway rats are known to live in social groups including both related and unrelated individuals. They recognise individuals by odour [21] and can discriminate different levels of relatedness in social partners [22]. Furthermore, they frequently show social behaviours such as allogrooming, joint aggression towards intruders, assemblage formation in winter and food storing [23]. In iterated prisoner's dilemma games, where two players can choose to either cooperate or to defect [10], they were shown to help an experimental partner based on direct and generalized reciprocity [18,19]. Both mechanisms can induce evolutionarily stable levels of cooperation [24-32]. In our experiments the focal test rats could produce food for a social partner present in an adjacent compartment by pulling a tray towards the cage (Figure 1). In the first experiment, social experience of the focal rats was manipulated by pairing them with cooperative or defective partners (stooges) that had either produced food for them ('cooperator') or not ('defector') in the identical but role-reversed situation (direct reciprocity [19]). We manipulated the costs of the helpful act by varying the resistance of pulling food for the experimental partner between 1 and 5 newtons, which covered the range from a very easy operation to a difficult action they could just accomplish. The rats were trained to recognise visual signals associated with the different degrees of resistance to enable them to decide about giving help dependent on costs already before handling the apparatus.

In the second experiment we manipulated the levels of need of social partners (stooges) by food restriction applied over night to induce hunger. In this experiment, the focal rats were given either a helping experience by an unknown third rat just before the test phase (generalised reciprocity [18]) using the same experimental setup, or they were not given a social experience (control). We tested for potential effects of the condition (body mass) of experimental partners and predicted that if their need is important for the decision of focal rats to help, potential receivers in bad condition should elicit more help than potential receivers in good condition.

We found that rats provide more help to cooperative than to defective partners, and that the amount of help provided decreases more strongly with increasing costs when the experimental partner is a defector. Furthermore, hungry rats received more help for food if they were light, whereas if the receiver was satiated, focal rats provided more help for heavy partners. Both results meet the assumptions for costs and benefits being important parameters in the decision of rats to help an unrelated social partner.

## Results

### Effect of cost

**Figure 2:**
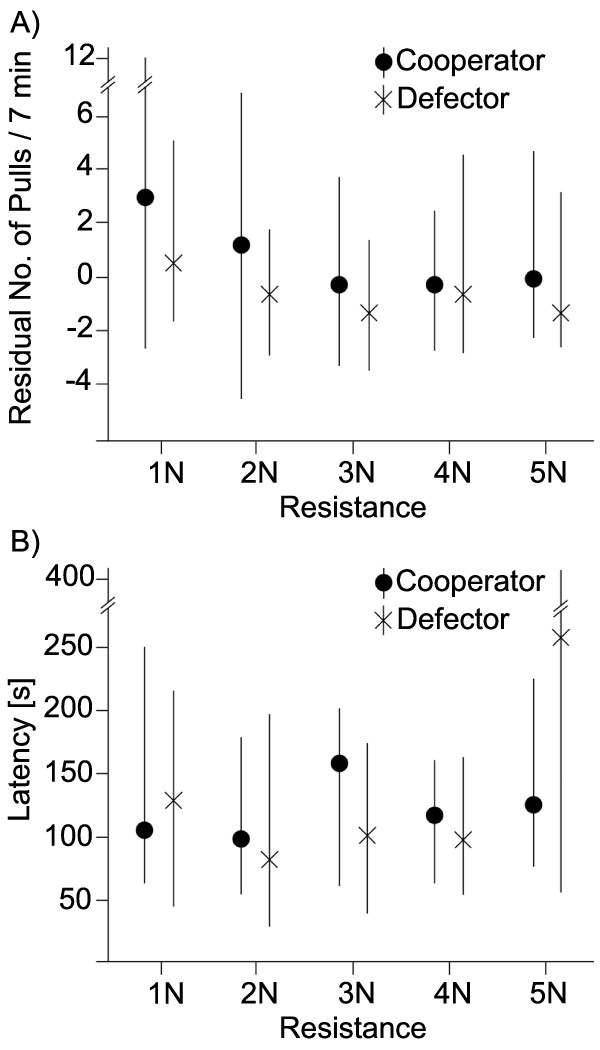
**Residuals of the number of pulls (A) and latency to first pull (B) in dependence of the pulling resistance and social experience**. (**A**) The frequency of pulling food for previous cooperators and previous defectors declined with increasing costs (measured as resistance in newtons (N); mean ± standard error; n = 14 rats tested in both situations). The focal rat pulled more often for cooperators than for defectors (*p *< 0.001; see text). (**B**) The latency from the start of experiment to the donor's first pull increased with costs when the focal rat was paired with a social partner that had previously defected (*p *< 0.001; mean ± standard error; n = 14).

In the first experiment the test rats pulled more often for a cooperator than for a defector (X_1_^2 ^= 16.990; *p *< 0.001). Further, the number of pulls decreased with increasing resistance in both social contexts (X_4_^2 ^= 38.547; *p *< 0.001). The latency until the first pull increased with increasing resistance when helping a defector, but not when helping a cooperator (X_4_^2 ^= 356.78; *p *< 0.001; Figure 2). When given the opportunity to acquire food for themselves, the rats pulled more often (X_2_^2 ^= 2793.9; *p *< 0.001) and with shorter time delays (X_2_^2 ^= 485.9; *p *< 0.001) than when pulling for a social partner.

### Effect of benefit

**Figure 3:**
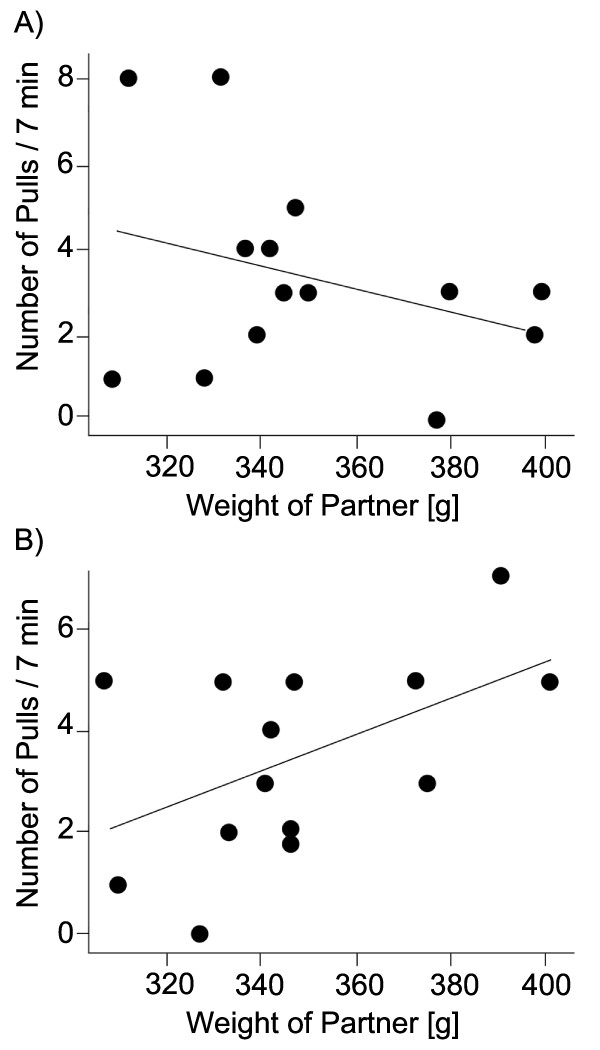
**Number of pulls in dependence of the weight of the social partner**. (**A**) The number of pulls of focal rats for a hungry partner decreased with increasing weight of the experimental partner (*p *= 0.013). (**B**) In contrast, rats pulled for satiated partners more often when these were heavier (*p *= 0.013; two overlapping points were slightly shifted to make them visible in the graph); n = 14 for both treatments.

In the second experiment the hunger status of social partners together with their condition (weight) influenced the number of pulls by the focal rat (X_1_^2 ^= 6.159; *p *= 0.013; Figure 3). When the receiver was hungry, focal rats pulled more often for light than for heavy partners. In contrast, when the receiver was satiated, focal rats pulled more often for heavy partners. When the focal rats had not been helped by a third rat before, they also pulled more often for heavy experimental partners than for light ones (X_1_^2 ^= 4.036; *p *= 0.044).

## Discussion

Focal rats pulled more often after receiving help than after receiving none, confirming that they applied direct (first experiment) and generalised (second experiment) reciprocity [18,19]. Apparently, the rats attempted to raise the probability of getting help back in the future by providing more help to cooperators than defectors. Thereby they took the costs of the helpful act into account, as the number of pulls decreased with increasing resistance, and the latency until the first pull increased only when helping a defector, but not a cooperator. In a self-rewarding control situation the test rats pulled more often and with a shorter time delay for themselves than they did when helping a social partner, which shows that the pulling does not merely reflect a conditioned response. One might argue that instead of reflecting a strategic decision, the reduction of the pulling frequency with increasing resistance could reflect limited physiological ability. However, this does not explain why the latency to start pulling increased with increasing workload. Obviously the costs of helping were assessed even before the rats experienced the burden, by using the visual cues they had been trained to relate to specific workloads (see Methods section). Previous studies of reciprocity in rats using the same experimental setup revealed that the intrinsic pulling frequency (the number of pulls a rat performs when alone in the cage, i.e., without social partner) did not differ after experiencing a cooperator or a defector [18]. Therefore, pulling in these experiments is clearly a social act and does not reflect copying, reinforcement or mere conditioning.

The results of our second experiment suggest that rats judge the relative need of their social partner and adjust their helping behaviour accordingly. Focal rats provided more food to hungry receivers in bad body condition that would suffer from hunger more severely. Two mechanisms might be responsible for the test rat's awareness of their experimental partner's need: 1) The donor may gain information about the hunger level through the signalling of need by the receiver. For example, rats may use ultrasound vocalisation as such signals are applied in a range of social and stressful contexts [33]. However, differences in activity and behaviour between hungry and satiated rats were not observed in our experiment. 2) The donor may judge the need of the recipient through inadvertent physical cues. For example, rats gain information about food preferences of group mates through odour cues from their breath [34]. They might also gain information about other, hitherto unknown traits from their experimental partner's body.

When the receiver was satiated, the focal rats pulled more often for heavy than for light partners, which was also the case when the focal rat had not received help before. This might suggest that unconditional help, in particular, is contingent on the relative status of a social partner, as in Norway rats body mass correlates with dominance [35]. Helping a dominant partner might reduce the probability of punishment for defection [36,37]. Alternatively, helping dominant individuals preferentially might be more effective in improving the social reputation of the donor than providing help to a subordinate partner [38].

## Conclusion

To our knowledge, our study provides the first empirical evidence for strategic decisions of helpers contingent on the costs of donors and the potential benefit to receivers in a prisoner's dilemma situation, which supports the predictions of theoretical models of the evolution of cooperative behaviour. In a different experimental situation, capuchin monkeys (*Cebus apella*) were found to be affected in their decision to invest toward collective goals by the relative effort that was demanded, i.e. the number of times a monkey had to hand a rock to an experimenter before getting a reward [39]. Hence, as predicted by evolutionary theory animals do seem to take costs into account when deciding about cooperation. This is consistent with the observation that examples of reciprocal cooperation in natural or semi-natural conditions often involve seemingly inexpensive behaviours such as allogrooming [14,40,41].

Furthermore, our data indicate that potential benefits to the receiver can also influence the propensity of animals to cooperate, as was suggested by the interaction between effects of experiencing cooperation and the need (hunger) and body condition of the social partner. At the proximate level, generosity of similar type has been assumed to be 'gratifying' to the donor in non-human primates [42], which might constitute a reward mechanism involved in the delivery of benefits to others. At the ultimate level, the probability to receive future help should be increased by preferably helping needy social partners for such discrimination mechanism to evolve. This can be accomplished in two possible ways, either if it raises the propensity of receivers (direct reciprocity) or eavesdroppers (indirect reciprocity) to help the cooperator on future occasions, or if it increases the survival probability or condition of needy receivers, which in turn would raise the probability that these can exert help to the cooperator (direct reciprocity) or to anyone (generalized reciprocity) in future interactions. As rats have been demonstrated to show both, direct and generalized reciprocity, both possibilities may apply [27].

In our experiment the motivation of test rats apparently changed between different contexts: When helping a hungry social partner after receiving help from someone else, the need of the partner rat influenced the motivation of the donor to help, which might indicate empathy [43]. In contrast, when the rat had not been helped before, its cooperation seemed to be motivated by other functions, such as punishment avoidance or building reputation. Factors that had been believed to be largely confined to human social behaviour may influence the behaviour of other social animals as well [44], indicating that reciprocal cooperation in animals and humans should be viewed in an evolutionary framework.

## Methods

### Housing and training of the rats

The female rats were bred in the Animal Physiology Department, University of Groningen, Netherlands and were housed at the Ethologische Station Hasli near Bern in groups of 3 - 5 individuals with their litter-mates. The climatic housing conditions were held constant at 20°C and 50-60% humidity under a 12:12 h light-dark cycle with lights on at 8 pm. Food (rat pellets) and water were provided ad libitum. As rats are nocturnal, the training and experiments were performed during the dark phase. The rats were given extensive handling experience from an early age, and therefore they were used to the presence of an observer. This also ensured that the rats were not stressed by the handling procedure and transportation during the training and the experiments. The experimental setup was similar to that used in previous cooperation experiments with rats [18,19]: A testing cage was divided by a wire mesh into two compartments. In front of the cage, a tray was installed that glided with ball bearings on a rail. A stick was attached to it so that it could be reached by the rat in the cage (see Figure 1). The rat pulling the stick moved the tray closer to the cage, so that a food reward (one oat flake) placed on the tray could be reached either by herself (early training) or an experimental partner (major training and all experiments).

During a 3-months training phase, the rats were trained individually to pull the stick. First, the rats were made familiar with the tray by finding the food reward on the tray without having manipulated the stick. Then the tray was moved stepwise out of the cage so that the rat had to pull the tray to reach the reward. The distance of the tray from the cage was increased continuously until the rat pulled the tray along the whole way without the help of the trainer. Rats that did not learn to pull at that stage were excluded from the study. In a second training phase, the resistance of the tray was increased manually from 1 - 5 newtons in steps of one with help of adjustable screws. This range of resistances was elaborated in a pilot study, where the rats were tested for the maximum resistance at which they could still pull the tray. Specific visual cues corresponding to the respective resistances were presented in front of the cage so that the rat could easily see them. By pulling the tray under varying resistances with corresponding symbols, the rats learnt to associate the visual cues with the workload. Rats had been shown previously to use visual signals for behavioural decisions [45]. Subsequently, the rats were made familiar with a reciprocity situation: Two unfamiliar, trained rats were placed in the cage separated by a wire mesh. Only one rat had access to the stick and could pull the tray towards the cage under the low resistance of 1 newton. Only the experimental partner, but not the pulling rat, had then access to the food reward. The roles were exchanged in the subsequent session. The number of pulls rats had to perform before switching roles was increased in every session until the rats kept their role as donor or receiver continuously for seven minutes.

The housing of the rats and the experimental procedure adhered to the Association for the Study of Animal Behaviour Guidelines for the Use of Animals in Research and were approved by the Swiss Federal Veterinary Office (Kanton Bern, permit #71/6).

### Experimental design

The experimental design and procedure was modified after [18,19]. For the experiment, a defective and a cooperative partner were assigned to each of 14 focal rats. The testing cage was the same as the training cage, divided in halves by a wire mesh. Every rat was put into one compartment of the cage with visual, acoustical and olfactory contact to its experimental partner. In the first experiment, every focal rat faced two different social situations in a random sequence: 1) she was helped by a cooperative partner ('cooperative situation') that pulled for the focal rat for a period of 7 minutes, during which at least one pull occurred (median = 4 pulls per 7 min); or 2) she did not receive help from the experimental partner (defective situation) during a period of 7 minutes, during which the latter did not pull the stick [18]. Directly after the experience phase, the stick and the food reward were switched to the other side and the focal rat had now the possibility to provide help for its experimental partner (direct reciprocity paradigm). The duration of this test phase was again 7 min, the resistance of the tray was set to a value of 1 - 5 newtons and the corresponding visual cue was provided in front of the cage. Both rats received a food reward directly after the experience phase to prevent pulling behaviour to be caused by reinforcement [18]. During the experimental phase, the acting rat was not directly rewarded for pulling. On a single day, all 14 rats were tested for both, a cooperative and a defective situation with the same pulling resistance, followed by a break of two days. The sequence of the two social experiences and five different resistances was randomised, whereas the cooperative and defective partners stayed the same throughout the experiment. Both, the frequency and latency to pull were noted and all the rats were weighed after each replicate. Increasing resistance of the tray reflects increasing costs of helping, while the latency to start pulling and the number of pulls performed by the focal rat during an experimental phase of seven minutes were used as measures of the propensity of test rats to help their social partner.

For the second experiment, the social partners were food restricted over night to induce hunger and were presented to the focal rats as partners in the same experimental setup but without changing the donors' workload, which was kept at 1 newton. The focal rats were given a cooperative experience by a third rat before having the opportunity to help the food restricted partner (generalised reciprocity paradigm [18]). In the control situation the focal individuals were not provided with any social experience, which allowed to test whether rats would provide unconditional help as readily as generalised reciprocity. The sequence of both, social experience and hunger status of the experimental partner were randomised, with every focal rat being tested in all four situations. The rats were not related to each other and had never met or interacted before. The latency and frequency of pulls were noted and the focal and partner rats were weighed after each replicate.

### Statistics

To test for treatment effects (social experience, hunger status, resistance), we used generalised linear mixed models, including individual identity of the focal rats as subject in all models, as the experiment used a repeated measures design. All tests employed correction for Poisson distributions of the data. As factors, we included the single treatments and their combinations for each experimental session. All analyses made use of the R statistical software (version 2.8.1, http://www.r-project.org) with the "lme4" and "lattice" packages.

## Competing interests

The authors declare that they have no competing interests.

## Authors' contributions

KS designed the experiments, trained the animals, collected and analysed behavioural and weight data, and wrote the manuscript. MD participated in the collection of weight data, training of the animals and designing of methodological details. MT devised, designed and supervised the study and wrote the manuscript. All authors read and approved the final manuscript.

## Authors' information

K.S. is currently a doctoral student investigating the evolution of the immune system of bats, M. D works on hormonal influences on cooperation in rats for her PhD, M. T. is professor of behavioural ecology studying the evolution of cooperation and alternative behavioural tactics.
